# Supplementary material for: Spectrum decomposition in Gaussian scale space for uneven illumination image binarization
Source: PLoS One. 2021 Apr 30;16(4):e0251014. doi: 10.1371/journal.pone.0251014 (PMC8087106; doi:10.1371/journal.pone.0251014)
Supplement: Attachment — Submitted filename: Response to reviewers.docx [file pone.0251014.s002.docx]

Dear editors and reviewers:

Thank you for your letter and the reviewers’ comments on our manuscript entitled “Spectrum Decomposition in Gaussian Scale Space for Uneven-illumination Image Binarization”(ID: PONE-D-20-30273). Those comments are very helpful for revising and improving our paper, as well as the important guiding significance to the other research. We have studied the comments carefully and made corrections which we hope meet with approval. The main corrections are in the manuscript and the responds to the reviewers’ comments are as follows.

Replies to the editors’ comments.

1. Using PLOS ONE's style requirements.

**Response:** Thank you for the style template provided in the email. We have changed the style according to the style templates, which includes equation naming and file naming, etc.

2. Improving language usage, spelling, and grammar.

**Response:** Thank you for your suggestions on the grammar and usage of the article. We used **AJE (American Journal Experts)**, a professional scientific editing service provider, to edit our manuscript, and the order number is GVXB238V. A copy of the manuscript showing changes is submitted in the supporting information, and to make it easier to show the changes, we added a page number in the footer.

3. Removing Funding Statement.

**Response:** We are sorry to mention the funding statement in the text, and it has been removed from our manuscript.

4. Referring to Figure 14 in text.

**Response:** Thank you very much for reminding about this. It has now been correctly referred in the text (marked in red on page 15), and due to the addition of the comparison experiment, Figure 14 has been split into Figure 14 and 15 for the convenience of display.

5. Referring to Tables 2 and 4 in text.

**Response:** We are sorry that such important data is not referred in the text, and thank you for your reminder about this. In supporting information, the reference to Table 2 is marked in red on page 15

, and Table 4 is marked in red on page 21.

6. About Data Availability statement.

**Response:** The test set part of this data set is collected by our team and manually annotated them, and we submit the test images as supporting information for Data Availability.

Replies to the reviewers’ comments.

Reviewer #1:

1. The related works/ literature is poor.

**Response:** Introduction has added some literatures about segmentation area from 2019 to 2020, which include one survey, and some recently traditional methods, deep learning methods. The survey can give us a lot of references. The Deep learning method use multi-scale and multi-level architecture, this structure can extract more information and feature from images.

2. Make the Fig 2 (Flowchart of all processes) clear.

**Response:** This has been revised.

3. Fig 7 and 8, consists of parts from a-h, it is recommended to explain each graph (a) represents what? b represents what?

**Response:** I’m sorry we did not clarify the intention of Fig 7 and 8. The two figures They represent two type of testing text images. Because of our experiments show that different methods have different sensitivity to images under different environmental brightness. In other words, some methods have better performance in brighter image while some or not. The legends of Fig 7 and Fig have related description. Therefore, we divide the images as brighter data and darker data.

In addition, we did not add the representation of a-h om Fig 7 and 8. Because the data are text images, all of them have rich borders and textures while having uneven lighting background.

Reviewer #2:

1. References used in the paper are not latest.

**Response:** Sorry for not being able to show recent related research in the article. To introduce the work in the field of image segmentation in recent years, the references have added some literatures about segmentation area from 2019 to 2020, which include one survey, and some recently traditional methods, deep learning methods. The survey can give us a lot of references. The Deep learning method use multi-scale and multi-level architecture, this structure can extract more information and feature from images. One of the newly added traditional methods is used for comparison.

2. Compare the proposed work with edge based and deep learning based techniques.

**Response:** Thank you for your suggestions, and we compared two edge-based methods, BSR [1] method and PFE [2] method. However, we just demonstrate the PFE in our paper, the performance of BSR is relatively poor on both text images and non-text images, we demonstrate some results in following Fig 1.

|   (**a**) |   (**b**) |
| --- | --- |
|   (**c**) |   (**d**) |

**Fig 1. BSR demonstration.** (**a**)(**b**) are input images, (**c**)(**d**) are segmented images.

The segmentation of PFE on text images is not satisfactory, but it does a good performance of segmentation on non-text images. For the PFE method, we first use the deep learning-based boundary detector HED [3] recommended by the PFE author’s [Github](https://github.com/chaoweifang/PFE) to detect the boundary. However, if detected boundary is not good, segmentation tend to perform poor, which are showed by Fig 2.

|   (**a**) |   (**b**) |   (**c**) |
| --- | --- | --- |
|   (**d**) |   (**e**) |   (**f**) |
|   (**g**) |   (**h**) |   (**i**) |

**Fig 2. PFE demonstration.** (**a**)(**d**)(**g**) are input images, (**b**)(**e**)(**h**) are boundary, (**c**)(**f**)(**i**) are segmented images.

As for the deep learning method, because our method is designed to segment images with unevenly illuminated under industrial conditions with a small amount of data, it is easy to overfit our data by using a deep network when the amount of test data is small. Therefore, we did not use deep learning methods for comparison.

References

1. P. Arbeláez, M. Maire, C. Fowlkes and J. Malik, "Contour Detection and Hierarchical Image Segmentation," in IEEE Transactions on Pattern Analysis and Machine Intelligence, vol. 33, no. 5, pp. 898-916, May 2011, doi: 10.1109/TPAMI.2010.161.
2. Fang C, Liao Z, Yu Y. Piecewise Flat Embedding for Image Segmentation. IEEE Transactions on Pattern Analysis and Machine Intelligence. 2019;41(6):1470–1485.
3. Xie S, Tu Z. Holistically-nested edge detection[C]//Proceedings of the IEEE international conference on computer vision. 2015: 1395-1403.
